# Diabetes Program at Schools in Turkey

**DOI:** 10.4274/Jcrpe.516

**Published:** 2012-06-09

**Authors:** Şükrü Hatun

**Affiliations:** 1 Kocaeli University Medical School, Department of Pediatric Endocrinology and Diabetes, Kocaeli, Turkey; +90 262 303 87 31sukruhatun@gmail.com

**Keywords:** Diabetes, children, school

## INTRODUCTION

In-tro-duc-ti-onIn Turkey, there are approximately 20 000 children with diabetes, who are predominantly at school age. Most of these children encounter problems in their educational environment and it is possible to solve the problems by means of a comprehensive program. Diabetic children should not be kept away from physical education lessons and activities such as school trips. On the contrary, they should be motivated to participate in all activities with their schoolmates. If possible, in schools with diabetic children, a nurse or a counselor should be trained in identification and management of symptoms of diabetes emergencies and a place should be established for measuring blood glucose and for injecting insulin. Refreshments as well as toilet breaks should be allowed during the classes. Diabetes nutrition should be provided at boarding schools. Moreover, teachers should learn the symptoms of diabetes and be able to recognize these children earlier in order to avoid dangerous events at diagnosis.

On the other hand, obesity frequency among the 6-16-year age group has increased from 5% to 10.5% (16.3% among the high-income level group) in the last 8 years in Turkey. At least 1/3 of these children have a greater risk of developing adulthood obesity and/or type 2 diabetes in the future. Childhood obesity results mostly from consuming high-calorie food known as “junk food” and sugar-sweetened beverages, in addition to eating fast and leading an inert lifestyle. Preventing obesity in adulthood depends mainly on the efforts in childhood and puberty. In recent years, much more attention is directed towards this issue, the Turkish Radio and Television Supreme Council has limited the advertisements for high-calorie food, and finally, the Ministry of National Education has restricted the sales of junk food and sodas at schools.

**Diabetes Program at School**

The Program is developed by means of a joint protocol initiated by the proposal of the Diabetes Working Group embodied in the Turkish Pediatric Endocrinology and Diabetes Society in cooperation with the Ministry of National Education and Ministry of Health. The program, unconditionally supported by Sanofi-Aventis, has three main objectives:1. Raising awareness about type 1 diabetes via school and teachers. Ensuring an early diagnosis of type 1 diabetes and decreasing the frequency of diabetic ketoacidosis among school children.2. Strengthening the diabetes care in children at school age and solving the problems these children experience.3. Creating healthy attitudes toward nutrition among school children and raising awareness about obesity.

**Actions Taken Until Now**

An awareness poster under the title “Is my child a diabetes patient?” is now on the walls of 60 000 schools across Turkey. It has reached approximately 650 000 teachers and 16 million school children at primary and middle schools. Access to a brochure on diabetes in childhood, a guide on child’s diabetic care at school and a presentation on the training of teachers has been provided on related web pages and an official letter has been sent by the Ministry of National Education to the Provincial Directorates of National Education about the actions to be taken within the scope of the program. Pediatric Endocrinology Clinics across the country began to send a standardized letter to diabetic children and their teachers containing a program guide on child’s diabetes care at school.A short film was prepared for teachers, and the web address www.okuldadiyabet.org is now launched. Diabetes Program at School was initiated on November 12^th^, 2010 in Istanbul with the participation of a large group of teachers and this meeting attracted big attention from the press. On April 1^st^, 2011, a workshop was organized for the development of the program and planning of the steps in the coming period. Educational material about Diabetes Program at School may be reached through the websites www.meb.gov.tr, www.sdb.meb.gov.tr, www.saglik.gov.tr,

www.beslenme.saglik.gov.tr,

www.cocukendokrindiabetes.org.tr,

www.arkadasimdiabetes.com

Training in SchoolsA workshop on diabetes and obesity is going to be organized this year in the week of November 14th, World Diabetes Day, by the Ministry of National Education at 60 000 schools within the scope of Diabetes Program at School. On October 10th, 2011, a one-day training meeting was organized in Ankara for preparation for the training at schools across the country, with the participation of 300 staff comprised of 2 authorities from the Ministry of National Education, 1 from the Ministry of Health, and experts on diabetes and obesity among children. Diabetes and obesity issues were mainly explained in that meeting, the necessity to implement a circular in school canteens was emphasized, and the way of organizing school trainings in the week of November 14th was explained. In addition, training and awareness films prepared for children were introduced.

School children (7 500 619 children) and teachers (583 182 teachers) joined the school trainings that are to be organized within the scope of Diabetes Program at School. Two films with a total duration of 24 minutes and explaining the issues related to diabetes and obesity among children were runned at all schools. A training platform is generated on the website www.okuldadiyabet.org for easy access to the educational material. At this platform, there are films, brochures and posters on diabetes and obesity aiming at continuation of training and raising awareness. The film about diabetes explains living with the disease with the words of a diabetic child and emphasizes the difficulties in school life and the expectations of diabetic individuals. Program effectiveness will be measured by means of information and awareness research studies before and after the trainings.

**Şükrü Hatun, MD, Professor of Pediatrics,**

**Coordinator of Diabetes Program at School**

**On behalf of the Childhood Diabetes Group at the Turkish**

**Pediatric Endocrinology and Diabetes Society**

